# Expression of semaphorin 3A and its receptors in the human intervertebral disc: potential role in regulating neural ingrowth in the degenerate intervertebral disc

**DOI:** 10.1186/ar2898

**Published:** 2010-01-05

**Authors:** Sotonye K Tolofari, Stephen M Richardson, Anthony J Freemont, Judith A Hoyland

**Affiliations:** 1 Tissue Injury and Repair Group, School of Clinical and Laboratory Sciences, University of Manchester, Stopford Building, Oxford Road, Manchester, M13 9PT, UK

## Abstract

**Introduction:**

Intervertebral disc (IVD) degeneration is considered a major underlying factor in the pathogenesis of chronic low back pain. Although the healthy IVD is both avascular and aneural, during degeneration there is ingrowth of nociceptive nerve fibres and blood vessels into proximal regions of the IVD, which may contribute to the pain. The mechanisms underlying neural ingrowth are, however, not fully understood. Semaphorin 3A (sema3A) is an axonal guidance molecule with the ability to repel nerves seeking their synaptic target. This study aimed to identify whether members of the Class 3 semaphorins were expressed by chondrocyte-like cells of the IVD addressing the hypothesis that they may play a role in repelling axons surrounding the healthy disc, thus maintaining its aneural condition.

**Methods:**

Human IVD samples were investigated using reverse transcription polymerase chain reaction (RT-PCR) to identify gene expression of sema3A, 3F and their receptors: neuropilins (1 and 2) and plexins (A1-4). Sema3A protein was also localised within sections of normal and degenerate human IVD and immunopositivity quantified. Serial sections were stained using PGP9.5 and CD31 to correlate semaphorin 3A expression with nerve and blood vessel ingrowth, respectively.

**Results:**

Sema3A protein was expressed highly in the healthy disc, primarily localised to the outer annulus fibrosus. In degenerate samples, sema3A expression decreased significantly in this region, although cell clusters within the degenerate nucleus pulposus exhibited strong immunopositivity. mRNA for sema3A receptors was also identified in healthy and degenerate tissues. CD31 and PGP9.5 were expressed most highly in degenerate tissues correlating with low expression of sema3A.

**Conclusions:**

This study is the first to establish the expression of semaphorins and their receptors in the human IVD with a decrease seen in the degenerate painful IVD. Sema3A may therefore, amongst other roles, act as a *barrier *to neuronal ingrowth within the healthy disc.

## Introduction

Chronic low back pain (LBP) is a widespread problem within the UK and epidemiological studies have shown that it affects approximately 50 to 80% of adults during their lifetime [1,2]. Low back pain may originate from various sources and is considered to be multifactorial. However, numerous studies using various imaging techniques, primarily magnetic resonance imaging, have shown that intervertebral disc (IVD) degeneration is one of the major underlying factors in chronic non-specific LBP, accounting for approximately 40% of all cases [3-6].

In the healthy adult, the IVD is largely avascular and aneural with sparse innervation and vascularisation to the outer lamellae of the annulus fibrosus (AF) [7]. During IVD degeneration a number of pathological processes occur that impact on the extracellular matrix constituents, the macroscopic and histological appearance, and ultimately the function of the IVD [8,9]. Evidence suggests that neoinnervation and neovascularisation may be integral steps in the pathogenesis of painful IVD degeneration [10-13] with particular interest focussed on the ingrowth of nociceptive nerve fibres and their association with chronic low back pain [12,14,15]. Yet, despite these studies the mechanism of neural and blood vessel ingrowth still remains an enigma although it is assumed that such ingrowth is stimulated or inhibited by a number of physiological factors.

Numerous studies have investigated factors that may stimulate neural ingrowth within the IVD. Immunohistochemical and *in situ *hybridisation techniques have demonstrated that endothelial cells accompanying nerves growing proximally into the degenerate IVD express neurotrophic factors such as nerve growth factor (NGF) [16]. Additional studies have also established the expression of NGF in native nucleus pulposus (NP) and AF cells which increases after stimulation with proinflammatory cytokines which have been identified in IVD degeneration [17]. Other research has identified the upregulation of NGF and brain-derived neurotrophic factor (BDNF) in degenerate discs when compared to a cohort of healthy samples [18]. Noteworthy is the evidence which suggests that neurotrophic factors may also induce nociception via upregulation of pain-related neuopepties such as substance P and calcitonin gene related peptide (CGRP) [19]. Other potentially stimulating factors for neural and vascular ingrowth include the proinflammatory cytokines IL-1 and TNF-α [17,18,20]. Additionally, mast cells have also been suggested as a stimuli for neural and vascular ingrowth [21]. Plieotrophin and vascular endothelial growth factor (VEGF) have also been shown to be expressed by disc cells which may contribute to neovasucularisation during disc degeneration [20,22,23].

In contrast to neural stimulation, an opposing mechanism may be a lack of inhibitory factors influencing encroaching nerves. There is a significant amount of evidence illustrating that chondroitin sulphated proteoglycans such as aggrecan, which is found at high concentrations within the NP, have the ability to inhibit neural and vascular ingrowth [24-28]. *In vitro *studies suggest that this inhibitory action is due to the direct effect of the aggrecan molecule, as it prevents formation and growth of axonal processes [28]. Aggrecan is known to become depleted in the degenerate IVD, and hence as aggrecan is lost, so are any inhibitory influences on invading nerves and blood vessels. However, apart from these studies into the inhibitory effects of aggrecan on neuronal extension into the IVD, there is little additional literature that investigates other potentially inhibitory factors to nerves in the IVD.

Axons are capable of navigating through the developing nervous system with extraordinary accuracy. This is partially due to the guidance of molecules such as semaphorins. Semaphorins, formerly known as collapsins, are a large family consisting of eight classes of secreted and membrane bound polypeptides that have been identified in both invertebrates and vertebrates. This family, Class 3 semaphorins (sema3), are a large subgroup of these polypeptides, containing members A-G, which are the only secreted form of semaphorins found in vertebrates. These semaphorins were originally characterised in the developing nervous system, dorsal root ganglion, peripheral and sympathetic nervous systems as chemorepellent molecules that acted to initiate axonal collapse in areas that they were found in high concentration, thus resulting in axonal guidance [29-31]. This phenomenon has been outlined in studies that have demonstrated that sema3A null mutant mice showed extensive defasiculation of peripheral nerves and branching of axons into territories normally avoided by healthy nerves [29], thus demonstrating that sema3A is important in controlling nerve growth by facilitated repulsion of the axonal growth cone. Although originally reported as being responsible solely for axonal guidance in the developing nervous system [30,31] recent evidence suggests that they have a more pleiotrophic nature, fulfilling many additional roles including regulation of angiogenesis, organogenesis, tumurogenesis, cell migration, cytokine release and immune modulation [32-35]. However, despite the increasing evidence base regarding the different functions of the semaphorins within the human body, from a review of current literature it seems that the complete *in vivo *biological role of these molecules still remains unclear.

There are two families of receptors to the Class 3 semaphorins that are usually found on developing axons, the first of which being the neuropilins (NRP) and the second being the plexins (PLX). The neuropilins consist of two members; NRP1 which is thought to be the principal receptor for sema3A and NRP2 which is thought to be the principal receptor for sema3F [36-39]. The mammalian plexins consist of nine members PLXA(1-4), PLXB(1-3), PLXC1 and PLXD1 [36-43]. Evidence suggests that the neuropilins lack a defined signalling ability themselves and must bind with plexins in order to form a multimeric *holoreceptor *signalling complex. This complex facilitates a signalling cascade which is thought to initiate rapid depolymerisation and endocytosis of F-actin, thus inducing cytoskeletal collapse of the axon [39,40,44]. Interestingly, as well as binding members of the PLX family the NRPs bind VEGF receptors to enhance VEGF_165 _activity suggesting they also play a role in regulating angiogenesis through the competitive inhibition of sema3A and VEGF against one another for overlapping binding sites on the NRP molecule [45,46].

Recent evidence has identified sema3A, 3F and a number of its receptors in multiple cell types during endochondral ossification [47]. Importantly, prehypertrophic and hypertrophic chondrocytes demonstrated positivity for the semaphorins which preceeded neurovascular invasion. As IVD cells are phenotypically similar to chondrocytes [48] we hypothesised that cells of the healthy IVD may express semaphorins as a means of repelling neurovascular invasion, and in disc degeneration the expression of semaphorins may be reduced causing a disinhibition of neural and vascular ingrowth. As such the aim of this study was to investigate the expression of semaphorin 3A and 3F and their receptors in the human IVD and to ascertain whether expression altered within different IVD regions or with severity of degeneration. IVD samples were also analysed by immunohistochemistry for PGP9.5 and CD31 to correlate semaphorin 3A expression with nerve and blood vessel ingrowth respectively.

## Materials and methods

Human IVD tissue was obtained at either post mortem or following surgery, with informed consent from the patient or relatives. Local research ethics approval (North West Research Ethics Committee) was obtained for this work. Post-mortem samples of normal and degenerate NP were obtained within 18 hours of donor death. Asymptomatic normal and degenerate discs obtained from donors at PM had no documented clinical history of LBP. Samples of degenerate NP were also obtained from patients, diagnosed by magnetic resonance imaging, who underwent disc replacement surgery or spinal fusion to relieve chronic low back pain. Patients suffering from classical sciatica were excluded from the study. All samples were obtained and processed as previously described in studies conducted by our group [49].

### Histological grading of NP tissues

To establish histological grade of degeneration NP samples were fixed in 4% paraformaldehyde/PBS and processed into paraffin wax. Five micron sections from the tissue blocks were cut and stained with haematoxylin and eosin and the degree of morphological degeneration graded according to previously published criteria [48]. The grading system generates a score between 0 and 12: a grade of 0 to 3 represents a histologically normal (non-degenerate) disc grades of 4 to 6 indicate mild degeneration, grades 7 to 9 moderate degeneration and grades 10 to 12 severe degeneration.

#### RT-PCR

A range of post-mortem and surgical samples were used, in which there were 14 samples (7 AF and 7 NP) in each cohort. The post mortem cohort ranged from aged 30 to 79 years (mean: 57 years) and the surgical cohort ages 29 to 56 years (mean: 39 years). Cells were isolated from each sample as previously reported [49] and RNA extracted using Trizol™ (Invitrogen, Paisley, UK) according to the manufacturer's instructions. RNA was then treated with 4U DNAse I (Invitrogen) to remove any DNA contamination and reverse transcribed using an Applied Biosystems (Warrington, UK) High Capacity cDNA Reverse Transcription kit as per the manufacturer's instructions.

Aliqouts of cDNA were subjected to PCR using primers specific for the house-keeping gene GAPDH, as well as sema3A, sema3F, NRP-1, NRP-2, PLXA1, PLXA2, PLXA3, PLXA4 and PGP9.5 (Table 1). Each 25 μl reaction mixture consisted of: 19.125 μl molecular biology grade water, 2.5 μl 10 × PCR buffer w/o MgCl_2_, 0.75 μl 50 mM MgCl_2_, 1 μl 5 mM dNTP mix, 0.25 μl of 100 μM respective forward and reverse primers, 0.125 μl Platinum Taq polymerase (Invitrogen) and 1 μl of respective cDNA (5 ng/μl). Articular cartilage cDNA and molecular grade water were used as positive and negative controls respectively. Amplification was performed in a thermal cycler (Veriti, Applied Biosystems, UK) under the following conditions: enzyme activation at 95°C for 90 seconds followed by 38 cycles of 95°C for 20 seconds, 60°C for 20 seconds and 72°C for 60 seconds; and a final extension step of 72°C for 10 minutes. PCR products were then run on a 2% agarose gel containing GelRed (Biotium, Hayward, CA, USA) alongside a 100 bp DNA ladder (Bioline Hyperladder IV, London, UK). Bands were visualised by UV transillumination and images acquired using Genesnap software (Syngene, Cambridge, UK).

**Table 1:** Human oligonucleotide primer sequences used for RT-PCR

Primer Name	Forward Primer	Reverse Primer	Amplicon Size (bp)	Genbank Accession Number
**GAPDH**	GCCTCCTGCACCACCAAC	GAGCTTGACAAAGTGGTCG	482	[Genbank: NM_002046]
**SemaA**	GAGACTTGGTATGATTTAGAAG	GCTCTGTGTCAATGACTTCC	618	[Genbank: NM_006080]
**SemaF**	AGTGTCCGTACGATCCCAAG	CAACAGTGACCACCGTCATC	328	[Genbank: NM_004186]
**NRP-1**	CTCCTGTTGTGTCTTCAGG	CCCGATGAGGATCGGATTC	396	[Genbank: NM_003873]
**NRP-2**	CATGCACTATGACACCCCTG	ATGGGTTCCATGCAGTTCTC	756	[Genbank: NM_201266]
**PLXA1**	GACTTCCTGCTGACCCTGAG	GACTTCAACCTGAAGCCAGC	689	[Genbank: NM_032242]
**PLXA2**	GCTACAAGAGCTGGGTGGAG	CTCTCGGCTTGAAGAACCAC	538	[Genbank: NM_025179]
**PLXA3**	CAGCAGATCGACTACAAGAC	GCCGTGTCAGGTAGATCTC	564	[Genbank: NM_017514]
**PLXA4**	TGTCAGGTGTCAACGAGAGC	ATACACCTGCTCCTTGGTGG	350	[Genbank: NM_020911]
**PGP9.5**	TGCTGAACAAAGTGCTGTCC	AAAGGCATTCGTCCATCAAG	508	[Genbank: NM_004181]

#### Immunohistochemistry

Immunohistochemistry for sema3A, PGP 9.5 and CD31 was conducted on cohorts of healthy and degenerate disc tissue. The healthy disc cohort consisted of 10 healthy samples which were histologically graded between 0 and 3 according to previously published criteria [48] and had an age range of 30 to 61 years (mean: 51 years). There were a further 10 samples considered to be mildly degenerate (grades 4 to 6) ranging from ages 30 to 74 years (mean: 53 years), a moderately degenerate cohort of 10 samples (grades 7 to 9) ranging from ages 28 to 79 years (mean: 61 years) and a severely degenerate cohort (grades 10 to 12) of 10 samples ranging from ages 22 to 78 years (mean: 41 years). The latter two cohorts primarily consisted of samples obtained during surgery. The immunohistochemistry protocol followed was as previously published [49,50]. Antigen retrieval was performed using: type II trypsin for sema3A (1 mg/ml for 20 minutes at 37°C), heated citrate buffer for PGP9.5 (10% v/v citrate buffer pH6.0 for 15 minutes at 95°C) and pepsin for CD31 (1 mg/ml in 0.2 M HCL for an hour at 37°C). Subsequently, the slides were washed with tris-buffered saline (TBS) and non-specific secondary antibody binding was blocked with 25% normal goat serum diluted in 1% bovine serum albumin (BSA)/TBS for sema3A & PGP9.5 and 10% normal goat serum diluted in 1% BSA/TBS for CD31. Each primary antibody was diluted in 1% BSA/TBS. Sema3A was applied at a 1:250 dilution, PGP9.5 at 1:20 and CD31 at 1:10 and incubated at 4°C overnight. Slides were then washed with TBS and the biotinylated secondary antibody was applied: goat anti-rabbit (sema3A) and goat anti-mouse (CD31/PGP9.5). Both antibodies were applied at a 1:300 dilution in TBS. Positive controls included rat brain for sem3A, human tonsil for CD31 and human brain for PGP9.5. Binding of the secondary antibody was disclosed with the streptavidin-biotin complex (Vector Laboratories, Peterborough, UK) technique with 3,3'-diaminobenzidine tetrahydrochloride solution (Sigma, Poole, Dorset, UK). Sections were counterstained with Mayer's haematoxylin (Raymond A. Lamb, East Sussex, UK), dehydrated, and mounted with Pertex (CellPath Plc, Powys, UK).

Quantitative analysis of staining was performed and for each section five random fields of view were observed in each morphologically distinct region of the disc (nucleus pulposus (NP), inner annulus fibrosus (IAF), outer annulus fibrosus (OAF)). Two hundred cells were counted per region and the percentage of positive cells was then calculated for each region of the IVD. Statistically, the data were non-parametric and analysed using the Wilcoxon matched pairs test when comparing data within regions of the disc. Additionally, the Mann-Whitney U test was used to compare data across varying grades of degeneration. Data were then plotted as mean ± standard errors to represent the 95% confidence intervals.

## Results

### Gene Expression of sema3A and 3F in IVD samples

**Figure 1:**
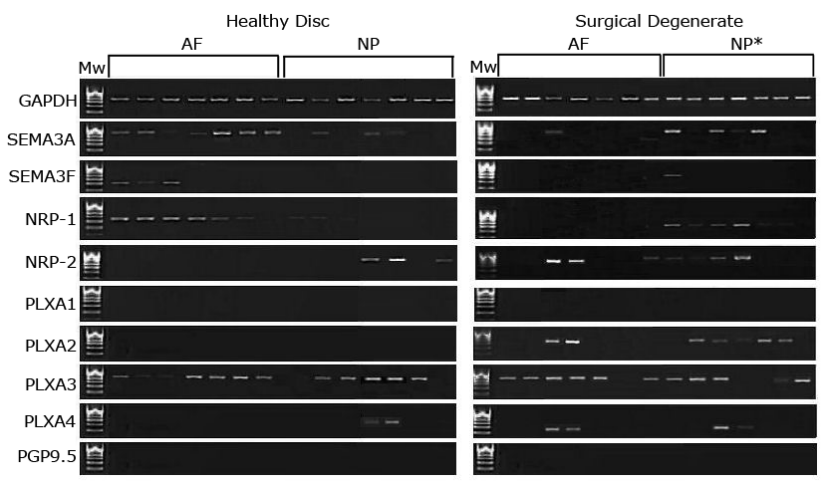
**Conventional RT-PCR for the house-keeping gene GAPDH, semaphorin 3A, 3F and their receptors**. This is a representative photograph following agarose gel electrophoresis of products from reverse transcription polymerase chain reaction for GAPDH, sema3A, sema3F, NRP-1, NRP-2, PLXA1, PLXA2, PLXA3, PLXA4 and PGP9.5. cDNA samples consisted of 14 healthy (non-degenerate) and 14 surgical (degenerate)intervertebral disc samples, with seven annulus fibrosus and seven nucleus pulposus in each. A 100 bp Hyperladder IV molecular weight marker (Mw) was included to allow estimation of correct band molecular weights. * represents samples assumed to be NP due to the nature of surgical intervention and sample collection technique.

Sema3A was expressed in 86% (6/7) of the healthy AF samples and 57% (4/7) of the healthy NP samples (Figure 1). Sema3F expression was observed in three (43%) of healthy AF samples and no expression was detected in the healthy NP samples. Within the surgical degenerate cohort sema3A was detected in only one sample with no sema3F expression detected in any degenerate AF samples. Sema3A expression was observed in three degenerate NP samples.

### Expression of receptor genes in IVD samples

NRP-1 was expressed in the healthy AF cells, but not by the NP. In degenerate samples expression was seen in six NP samples with no expression in AF samples (Figure 1). NRP-2 expression was observed in both healthy and degenerate NP samples with four healthy NP, degenerate AF and degenerate NP samples demonstrating positivity, albeit at differing levels. PLXA1 expression was not detected in any sample. PLXA2 was not detected in the healthy disc, but expressed in the degenerate disc. PLXA3 was expressed widely across all regions in both the healthy and degenerate disc and was found to be positive in 80% of samples. PLXA4 was expressed in two degenerate AF and NP samples with little expression in healthy IVD tissues. No PGP9.5 expression was observed in any of the samples.

### Immunohistochemistry

#### Immunohistochemical localisation of sema3A

**Figure 2:**
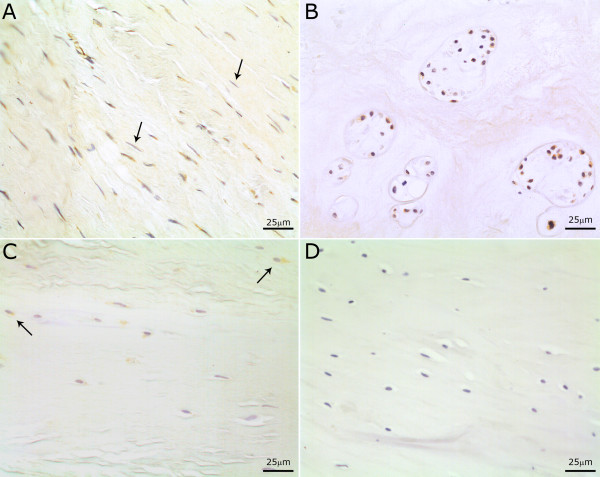
**Immunohistochemical localisation of sema3A in the healthy and degenerate disc**. **(a) **Healthy outer annulus fibrosus with cells demonstrating immunopositivity for sema3A with approximately 80% of the elongated AF cells being positive. Arrows highlight negative AF cells. **(b) **Degenerate NP demonstrating immunopositivity in the majority of chondrocyte-like cell clusters. **(c) **OAF in severely degenerate disc demonstrating a low immunopositivity. Arrows highlight positive AF cells. **(d) **Healthy OAF with the sema3A antibody omitted used as a negative control.

Immunoreactivity to the sema3A antibody was clearly identifiable throughout the entire cohort of IVD samples. Staining was primarily intracellular although some peri-cellular staining was also observed. Both the fibroblast-like cells of the AF and the chondrocyte-like cells of the NP stained positively (Figure 2). Immunoreactivity was also observed in vascular endothelial cells. In addition, the smooth muscle cells in the tunica media of small blood vessels, located primarily in the OAF of the more degenerate disc samples (grades 9 to 12) also exhibited positive staining. Of note, smooth muscle and endothelial cells were not counted for statistical analysis with only the chondrocyte-like cells of the NP and AF considered as sema3A immunopositive positive cells. There was no significant staining within the extracellular matrix of any of the disc samples.

#### Immunohistochemical localisation in the healthy disc

**Figure 3:**
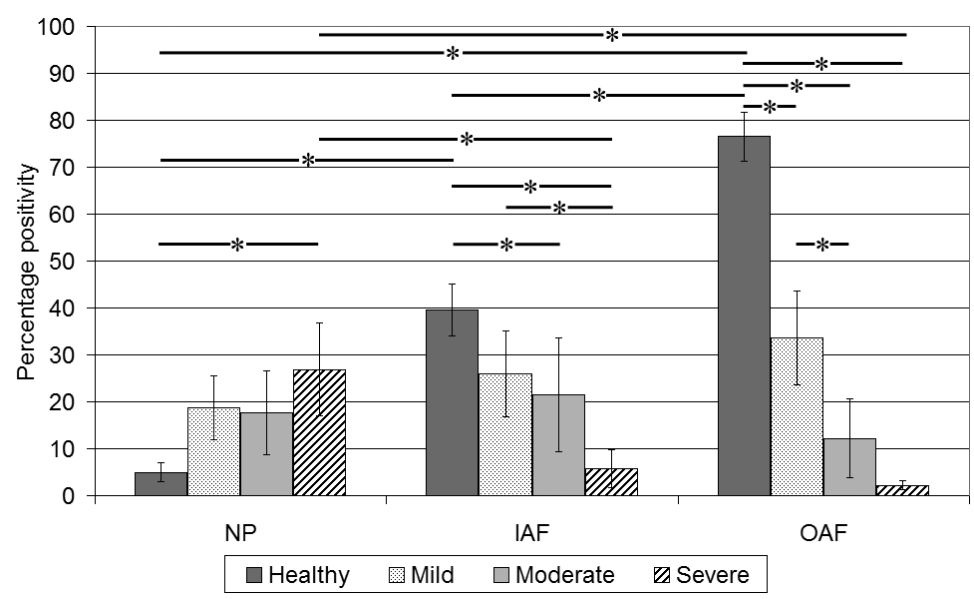
**Histogram illustrating the percentage of cells positive for sema3A per morphological region and stage of degeneration**. Values are mean ± SEM. *P < 0.05.

Immunopositivity was localised primarily to cells within the AF with approximately 80% of cells demonstrating positivity for sema3A (Figure 3). A gradient of expression was observed more proximally in the disc as the cells of the IAF showed 40% positivity and the NP cells only showed 5% positivity. There were significant differences (*P *≤ 0.01) in the number of cells demonstrating positivity for sema3A between all three morphological regions of the IVD (Figure 3).

#### Immunohistochemical localisation in the degenerate disc

Expression of sem3A in the OAF decreased significantly in the mildly degenerate cohort (grades 4 to 6) compared to that of healthy OAF (*P *≤ 0.01) (Figure 3). Percentage cell immunopositivity for sema3A was 33% in the mildly degenerate OAF compared to 26% in the IAF and 19% in the NP, although these differences were not significant (*P *≥ 0.05).

In moderately degenerate disc samples, immunoreactivity decreased to 12% in the OAF, 21% in the IAF and increased to 18% in the NP when compared to mildly degenerate samples. This positivity was found almost exclusively in cell clusters of the NP. No significant differences were found between the regions of the IVD. However, there were significant differences (*P *≤ 0.05 and 0.005) between the IAF and OAF of the moderately degenerate and healthy disc respectively (Figure 3). The severely degenerate disc samples showed a reduction in the number of sema3A immunopositive cells compared to the healthy disc with the OAF and IAF having 2.2% and 5.8% immunopositive cells respectively, which were significantly lower than expression in the healthy disc (*P *≥ 0.001 and *P *≥ 0.005). The number of sema3A immunopositive cells in the NP was significantly increased (*P *≥ 0.05) when compared to cells in the healthy disc with approximately 30% of cells (chondrocyte-like cell clusters) staining positively (Figure 2). Significant differences were also seen between regions of the disc (*P *≥ 0.05).

**Figure 4:**
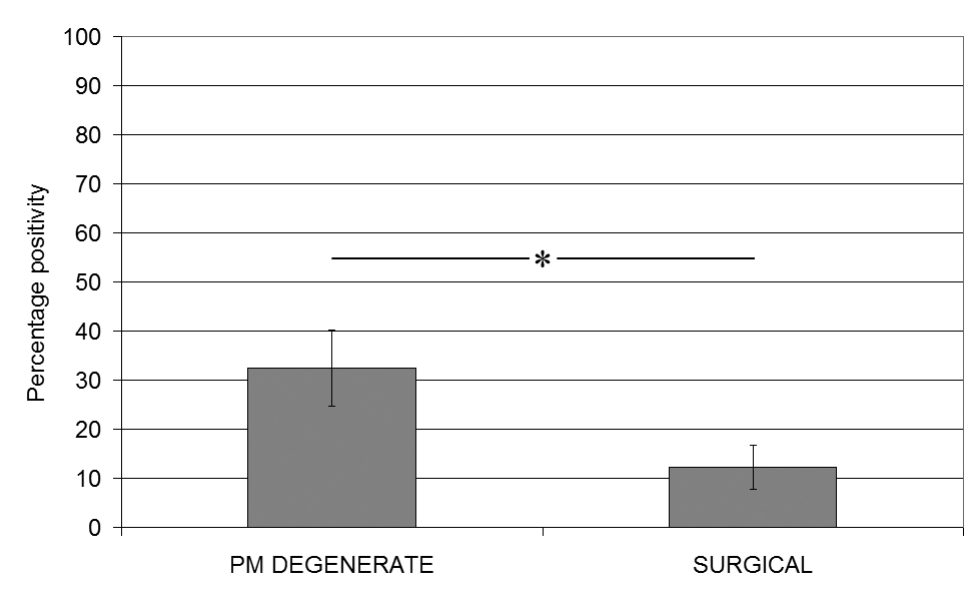
**Histogram illustrating the percentage of cells positive for sema3A in post-mortem degenerate samples and surgical degenerate 'painful' samples**. Values are mean ± SEM. *P < 0.05.

The degenerate sample cohort was further analysed to assess any differences in numbers of immunopositive cells between post mortem degenerate and surgical (symptomatic patients) degenerate samples (Figure 4). The average total disc sema3A cell immunopositivity in the PM degenerate cohort was 32%, while in the surgical degenerate cohort it was 12%. This reduction in sema3A expression was significant (*P *≤ 0.05). When comparing the surgical degenerate cohort to the healthy samples, which showed an average 50% cell immunopositivity, a markedly significant difference was observed (*P *≤ 0.001) (data not shown).

#### Immunohistochemical localisation of PGP9.5

**Figure 5:**
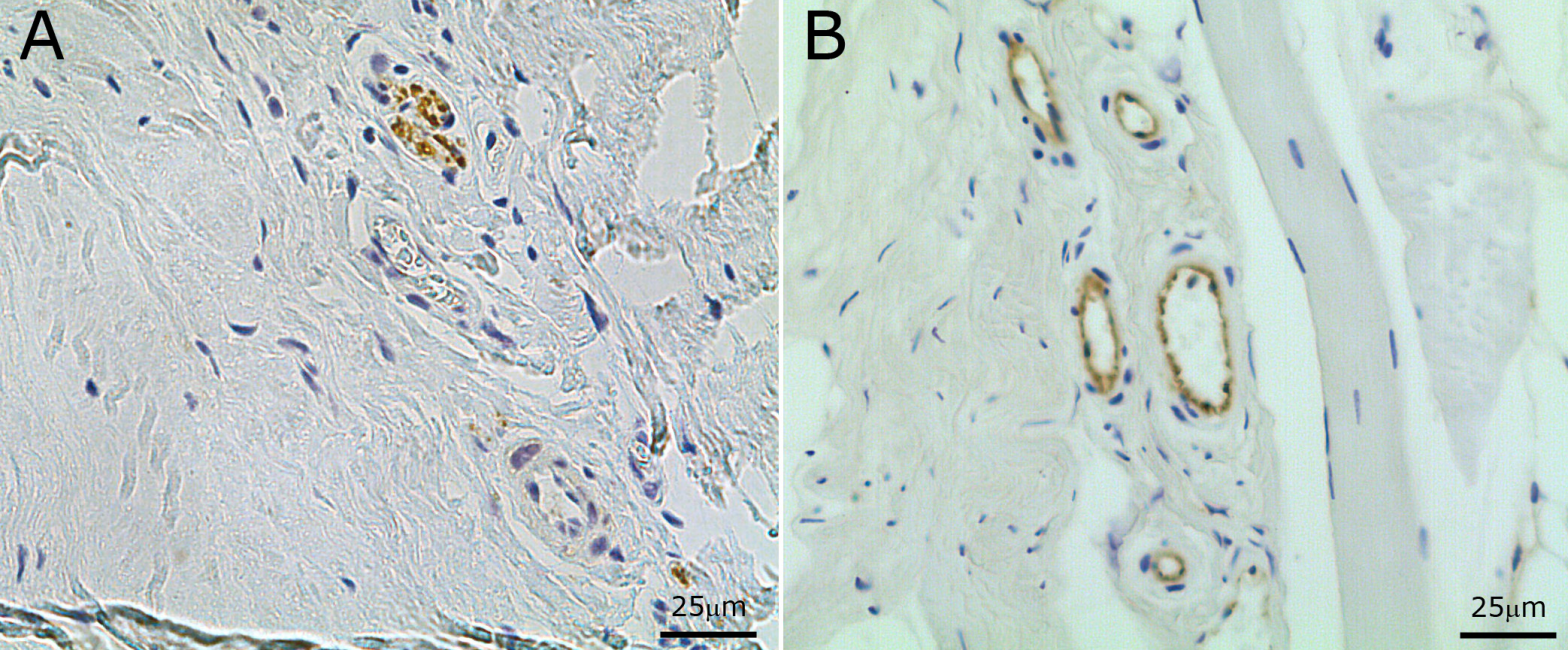
**Immunohistochemical localisation of CD31 and PGP9.5 staining in the disc**. **(a) **OAF of degenerate disc with neurovascular ingrowth staining positive for PGP9.5. **(b) **OAF illustrating blood vessel ingrowth in the degenerate disc, demonstrating positive endothelial cell staining for CD31.

The staining within the disc was sparse and specific to neuronal cells (Figure 5) with no positivity within the extracellular matrix of the disc. Immunopositivity was mainly localised to the areas of the OAF and IAF. Staining was intracellular, but had a granular appearance. Of the healthy cohort, 20% (2/10) of IVD samples demonstrated positivity while 8% (1/12) of PM degenerate samples showed areas of immunopositivity. Of the surgical degenerate samples 39% (7/18) showed positivity for PGP9.5.

#### Immunohistochemical localisation of CD31

Endothelial cells found within the disc were immunopositive for CD31 with staining found exclusively in endothelial cells surrounding developing blood vessels (Figure 5). The chondrocyte-like cells of the AF and NP were negative. No staining was observed within the extracellular matrix of the respective regions of the disc. Of the healthy samples only 20% (2/10) demonstrated positivity for CD31, 0% of the PM degenerate samples were positive, while 56% (10/19) of the surgical samples showed some level of positivity. Of note, five of the 10 CD31 positive samples were also positive for PGP9.5. Interestingly, the surgical degenerate cohort showed the lowest level of sema3A immunopositivity.

## Discussion

Evidence suggests that up to a third of adults in the UK suffer from chronic back pain at any one time [51]. The mechanism behind development of discogenic pain is not fully understood. However, nociceptive nerve fibres have been described within degenerate IVDs and there is developing evidence supporting their role in discogenic low back pain [14,16,18]. Although several studies have reported the involvement of stimulatory factors in regulating neural ingrowth in the IVD [17-19,21,22], the role of potential axonal inhibitory factors, other than aggrecan [27,28], has not been studied.

Our study is the first to localise expression of sema3A (an axonal inhibitory molecule) in the IVD and as such we have established a distinct regional variation in expression throughout the disc. We also observed changes in expression patterns during degeneration. Our immunohistochemical data demonstrated a high expression of sema3A in the cells of the OAF (approximately 80%) in the healthy disc which steadily declined towards the NP (5%). From current literature it appears that sema3A may act primarily as a chemorepellent factor against neurons and blood vessels and recent evidence suggests that sema3A is expressed by prehypertrophic and hypertrophic chondrocytes during endochondral ossification which precedes neurovascular invasion [47]. Our gene and protein expression results would therefore suggest that sema3A acts as a repellent to neurons in the healthy IVD and is expressed highly in the OAF as a potential obstruction to neural ingrowth.

Immunohistochemistry (IHC) data demonstrated that with degeneration there was a reduction in the total number of sema3A immunopositive cells with the severely and surgical degenerate samples having the lowest number of immunopositive cells. Areas of the degenerate OAF were particularly deprived of sema3A expression in comparison to the healthy disc, demonstrating a significant difference between PM normal and surgical degenerate or *painful *samples. Of note, our IHC findings demonstrated an increased PGP9.5 and CD31 expression in the IAF and OAF of the surgical degenerate samples in comparison to the healthy, indicating that more neuronal and endothelial cell types were present in these samples. Again, this supports our hypothesis that as expression of sema3A is reduced with degeneration, there is a disinhibition of neural and vascular ingrowth.

CD31, an endothelial marker, was expressed most highly in surgical degenerate disc samples localised primarily to the OAF and IAF. This suggests that the surgical samples were infiltrated with blood vessels to a high degree. Sema3A and isoforms of VEGF share the same co-receptor, the neuropilins [45,46]. Sema3A and VEGF compete for overlapping binding sites on the NRPs and therefore competitively inhibit one another [52]. We have shown that as the disc degenerates sema3A expression is reduced. This reduction in sema3A essentially withdraws the competition against VEGF and may therefore allow angiogenesis to occur as a result of VEGF binding. Conversely, VEGF has also been described as a survival factor for cells of the NP as it is expressed highly in hypoxic conditions [53]. As the disc degenerates and the cartilaginous endplates become calcified the disc tissue supposedly becomes hypoxic. These hypoxic conditions may be a driving force for VEGF upregulation which may cause inhibition of sema3A activity through competitive receptor binding.

In the healthy disc, very little sema3A was expressed in the NP, however with degeneration this trend changed and the NP became the predominant region of sema3A gene and protein expression in more degenerate samples. The IHC data demonstrated that this NP expression was localised almost exclusively to cell clusters. Clustering of these chondrocyte-like cells in the nucleus is a known characteristic of degenerate disc tissue and is thought to be due to increased cellular proliferation [54]. Sema3A has been investigated concerning its role in the modulation of cell proliferation [55]. Therefore there may be a potential role for sema3A in modulating the proliferation of cells in these clusters. Alternatively this increase in expression during degeneration may be part of a repair or rescue mechanism to prevent neural ingrowth into the delicate NP region of the disc.

Data provided from the RT-PCR results have shown that the disc cells express a number of candidate receptors for the Class 3 semaphorins. For example, sema3A has a high affinity for NRP-1 and NRP-1 was expressed highly by the disc cells as well as PLXA3. This suggests that sem3A may be secreted by a healthy disc cell and then bind to a NRP-1 molecule on the same cell or a neighbouring cell. The *in vivo *biological roles of the semaphorins are not fully understood, however sema3A plays a role in cell adhesion and migration [32,39], so there is potential that sema3A and to a lesser extent 3F may play a role in cell migration and distribution in the matrix. In support of this postulation, PLXA3 transfected MDCK cells have been shown to repel surrounding mesenchymal cells [56,57]. A similar phenomenon may occur in the IVD, which may be a reason that IVD cells are found so sparsely in the NP.

## Conclusions

From the evidence provided by this study we can conclude that sema3A is indeed expressed by cells of the healthy IVD, primarily OAF cells. This expression decreases with IVD degeneration and of note disc samples that have already exhibited neural ingrowth show the lowest expression of sema3A in comparison to healthy samples. The regulation of neurovascular growth into the degenerate intervertebral disc is quite clearly multifactorial as evidenced by a number of studies investigating different potential mechanisms for this phenomenon. Of note, it may not just be neurotrophic or chemorepellent factors that play a role in nerve growth regulation, but it may also be the complex alterations in the biochemical constituents of the extracellular matrix occurring during disc degeneration that influence neurovascular development/ingrowth in the disc. Previously mentioned studies [25,26] have discussed potential causative mechanisms for neural ingrowth regarding matrix degradation and have emphasised the role of molecules such as aggrecan in the regulation of neuronal growth. However, from evidence provided by our study, we can postulate that sema3A is expressed in high levels in the OAF of the disc as an additional or alternative means of preventing neural ingrowth, and as degeneration progresses we see a loss of inhibition and therefore the growth of invading nociceptive neurons into the otherwise aneural IVD. We propose that this may be a potential mechanism for the pathogenesis of chronic low back pain.

## Abbreviations

AF: annulus fibrosus; BDNF: brain-derived neurotrophic factor; BSA: bovine serum albumin; cDNA: complementary deoxyribonucleic acid; DAB: 3,3'-diaminobenzidine tetrahydrochloride solution; EDTA: ethylene diamine tetraacetic acid; GAPDH: glyceraldehyde 3-phosphate dehydrogenase; H&E: haematoxylin & eosin; HCL: hydrochloric acid; IAF: inner annulus fibrosus; IHC: immunohistochemistry; IL-1: interleukin-1; IMS: industrial methylated spirit; IVD: intervertebral disc; LBP: low back pain; MDCK: Madin-Darby canine kidney; NGF: nerve growth factor; NP: nucleus pulposus; NRP: neuropilin; OAF: outer annulus fibrosus; PCR: polymerase chain reaction; PLX: Plexin; RT-PCR: reverse transcription polymerase chain reaction; SEMA3: semaphorin class 3; TBS: tris-buffered saline; TNF-α: tumour necrosis factor; VEGF: vascular endothelial growth factor.

## Competing interests

The authors declare that they have no competing interests.

## Authors' contributions

SKT performed all tissue sample preparation, RT-PCR studies, data analysis, and drafted the manuscript. SMR participated in the study design and coordination, analysis of results and helped to draft the manuscript. AJF participated in analysis of results. JAH conceived the study, secured funding, participated in its design and coordination, analysis of results and co-wrote the manuscript. All authors read and approved the final manuscript.
